# Implementation and findings on a one-minute sit-stand test for prehospital triage in patients with suspected COVID-19—a pilot project

**DOI:** 10.1186/s12873-022-00605-9

**Published:** 2022-03-31

**Authors:** Julie Kjerulff, Allan Bach, Ulla Væggemose, Søren Helbo Skaarup, Morten Thingemann Bøtker

**Affiliations:** 1 Prehospital Emergency Medical Services, Research and Development, Central Denmark Region, Olof Palmes Allé 34, 2.floor, 8200 Aarhus N, Denmark; 2 Emergency Department, Regional Hospital Horsens, Sundvej 30, 8700 Horsens, Denmark; 3 Prehospital Emergency Medical Services, Ambulances and Physician Critical Care Unit, Central Denmark Region, Olof Palmes Allé 34, 2.floor, 8200 Aarhus N, Denmark; 4 Department of Respiratory Medicine and Allergy, Aarhus University Hospital, Central Denmark Region, Palle Juul-Jensens Boulevard 99, 8200 Aarhus N, Denmark; 5 Department of Anesthesiology and Intensive Care Medicine, Regional Hospital Randers, Skovlyvej 15, 8930 Randers, Denmark

**Keywords:** Prehospital Emergency Medical Services, Exercise testing, Corona virus disease 2019

## Abstract

**Introduction:**

During the initial Coronavirus Disease 2019 (COVID-19) pandemic wave, sparse personal protection equipment made telephone triage of suscpeted COVID-19 patients for ambulance transport necessary. To spare resources, stable patients were often treated and released on-scene, but reports from Italy suggested that some later detoriated. We implemented a prehospital sit-stand test to identify patients in risk for detoriation.

**Methods:**

The test was implemented as part of a new guideline in stable suspected COVID-19 patients younger than 70 years with no risk factors for serious disease triaged by general practitioners to ambulance response in the Central Denmark Region. Data were collected from April 6^th^ to July 6^th^ 2020. The primary outcome for this study was the proportion of patients treated with oxygen within 7 days among patients decompensating vs patients not decompensating during the test.

**Results:**

Data on 156 patients triaged to ambulance response by general practioners were analysed. In total 86/156 (55%) were tested with the sit-stand test. Due to off-guideline use of the test, 30/86 (34.8%) were either older than 70 or had risk factors for serious disease. 10/156 (6%) of patients had a positive COVID-19-test. In total, 17/86 (20%) decompensated during the test and of these, 9/17 (53%) were treated with oxygen compared to 2/69 (3%) in patients who did not decompensate (*p* < 0.001).

**Conclusion:**

In a population suspected of COVID-19 but with a low COVD-19 prevalence, decompensation with the sit-stand test was observed in 20% of patients and was associated with oxygen treatment within 7 days. These findings are hypotheses-generating and suggest that physical exercise testing may be usefull for decision making in emergency settings.

**Supplementary Information:**

The online version contains supplementary material available at 10.1186/s12873-022-00605-9.

## Introduction

Coronavirus Disease 2019 (COVID-19) rapidly turned into a worldwide pandemic [1, 2] and caused massive resource consumption that in some regions exceeded the maximal capacity of the health care system [3, 4].

COVID-19 disease can lead to acute lung injury, which necessitates hospital admission and oxygen therapy, according to initial reports in approximately 20% of patients [5, 6]. The majority of patients, approximately 80%, have a mild presentation and do not require neither hospital admission nor supplemental oxygen [7, 8]. Due to sparse personal protective equipment in Denmark during the initial pandemic wave (February 2020), general practioners (GPs) were prevented from physical examination of patients and telephonic assessment of suspected COVID-19 patients became necessary. Decision-making on whether to triage for ambulance transport was in this period thus based merely on a telephone interview of the patient or next-of-kin. On ambulance arrival, a large proportion of patients presented with stable vital signs and in order to reserve hospital and ambulance resources, stable patients without risk factors for serious disease were often treated and released on-scene. However, reports spreaded from Italy that seemingly stable COVID-19 patients not initially admitted to hospital later developed severe respiratory failure—a feature caused by cytokine storm phase [5, 6]. It was also reported that physical exercise testing could be used to identify the group of seemingly stable COVID-19 patients, who would later deteriorate.

The one-minute sit-stand test is a functional exercise capacity test used to evaluate physiologic reserves in patients suffering from lung disease [9–12]. It correlates with the well validated six-minute walk test, but contrary to the six-minute test, it is feasible in a prehospital setting [11, 13]. We implemented the sit-stand test as part of a clinical guideline for management of suspected COVID-19 patients in ambulances in the Central Denmark as a precautionary measure before treating and releasing seemingly stable low-risk patients on-scene. The aim of this study was to examine, if decompensation during a one-minute sit-stand test is useful for identification of seemingly stable patients suspected of COVID-19, who would later develop a need for oxygen treatment.

## Materials and methods

The study was a quality assurance project with prospectively collected data on the implementation of and results of a sit-stand test on patients with suspected COVID-19 receiving an ambulance in the Central Denmark Region during the intial pandemic wave (from 6^th^ April 2020 to 6^th^ July 2020). According to Danish ethical and legal regulations, informed consent from the patients was not required for data-collection for this quality assurance project (this waiver was approved by the Central Denmark Region Committees on Health Research Ethics).

### Setting and implementation

Central Denmark Region covers an area of 13,053 km^2^ and 1.3 million inhabitants corresponding to 23% of the Danish population [14]. Patients were triaged for ambulance transport either by GP via telephone interview, visit in GP clinic or GP home visit or following a citizen call to the emergency 1–1-2 number where medical personnel at the emergency medical communication center (EMCC) undertook criteria-based triage [15].

According to guidelines implemented before the COVID-19 pandemic, patients who had been triaged for ambulance transport after physical examination by a GP were not to be treated and released on-scene by ambulance personnel, whereas patients triaged after a telephone consultation only (GP or 1–1-2 call) could be treated and released after consultation with a physician [16].

**Fig. 1:**
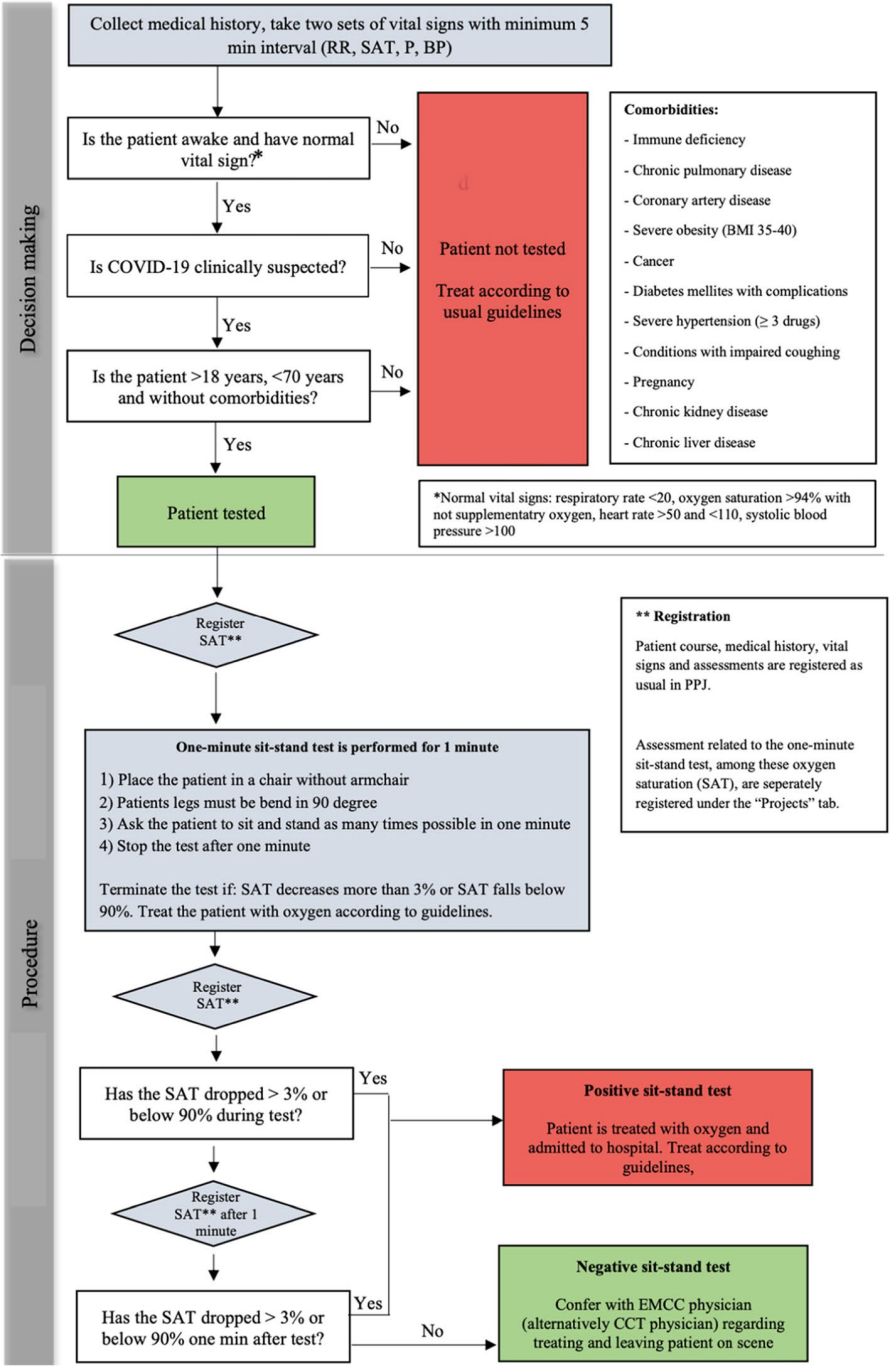
Sit-stand-test decision-making flow-chart (English translation)

During the initial pandemic wave, when GP’s were prevented from physically examining suspected COVID-19 patients due to lack of personal protective equipment, a new guideline for prehospital management of telephone triaged patients was implemented. According to this guideline, patients with known risk factors for progression to serious disease (i.e. age > 70, significant comorbidities and/or unstable vital parameters) were to be taken to hospital for further evaluation [17]. In stable patients < 70 years of age with no risk factors for serious disease, the one-minute sit-stand test was implemented as precautionary security measure before considering treating and releasing the patient on-scene (English translation of decision-support flow-chart in Fig. 1). Implementation of this new guideline was conducted according to organizational routines, as a written guideline distributed throughout the organisation. The guideline was supplemented by an instructional video describing which patients should be tested and how to perform the one-minute sit-stand test. Patients could at any time deny participation in exercise test.

### Exercise testing and clinical decision-making

The one-minute sit-stand test was conducted by the ambulance personnel on-scene according to Fig. 1. Prior to the test, all patients had to have two sets of normal resting vital signs (pulse, respiration rate, blood pressure and oxygen saturation) measured within a five-minute interval. The one-minute sit-stand test began with placing the patient on a chair without armrest, with the legs in a 90-degree bend. The patient was asked to sit and stand as many times as possible during one minute without supporting with the arms. The oxygen saturation was monitored by pulse oximetry during the test and continuously one minute after completion of the test. The one-minute sit-stand test was considered to indicate decompensation (i.e. positive test) if the oxygen saturation decreased > 3 percentage points and/or if the patient could not perform more than 12 sit-stand repetitions in one minute. The test was terminated and also considered positive if saturation fell below < 90% or if the patient was unable to continue the test [10, 18, 19].

Following completion of the sit-stand test, ambulance personnel telemedically consulted with a physician for patient triage. The decision on whether to admit the patient to hospital or not was made by the physician.

### Data collection

We collected data for this quality assurance project on all patients with suspected COVID-19 triaged to an ambulance response by a GP in the Central Denmark Region during the study period. Patients were identified through the electronic prehospital patient record (PPJ). Patient related data (age, gender, COVID-symptoms, comorbidities, vital parameters, prehospital and hospital treatment, COVID-19 status and final diagnosis) were collected by the primary investigator from PPJ and from electronic in-hospital patient records. For patients tested with the sit-stand test, the result of the test was entered into PPJ by ambulance personnel on-scene. Study data were collected and managed by the primary investigator using REDCap electronic data capture tools (REDCap, 10.0.10, Vanderbilt University).

### Outcome measurements

The primary outcome for this study was the proportion of patients treated with oxygen within 7 days among patients decompensating vs patients not decompensating during the test. Secondary outcomes were 1) the proportion of patients admitted to intensive care unit within 7 days in patients decompensating during the one-minute sit-stand test vs patients not decompensating, 2) the proportion of patients sit-stand tested before being treated and released on-scene and 3) the proportion of patients treated and released on-scene who were subsequently admitted to hospital, treated with supplementary oxygen, and/or were admitted to an intensive care unit.

### Statistical analysis

We lacked key knowledge on the patient group, size of the group, number of patients decompensating and number of these who would need oxygen supplement. Thereby, the study was exploratory and sample size calculation was not performed. Patients were included during the ongoing pandemic and duration of the study depended on the duration of the initial pandemic wave.

Binary data were analyzed by Chi^2^-tests and continuous data were examined for normal distribution and analyzed with students t-test. The level of significance was set at 0.05. Analyzes were performed using Stata 15 (StataCorp LCC, College Station, TX, USA).

## Results

### Baseline characteristics

**Fig. 2:**
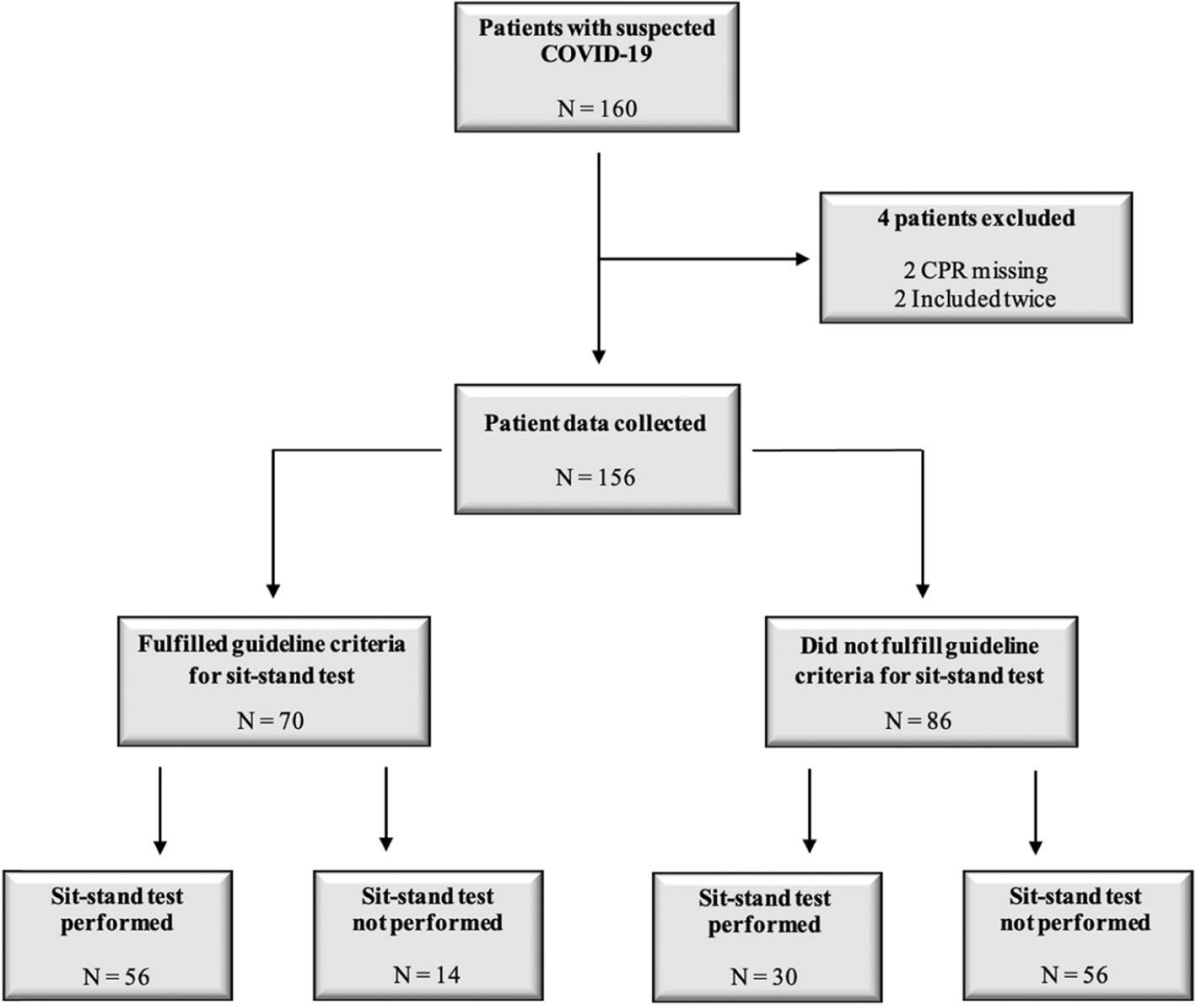
Flowchart of patients included in the study**.** The flowchart depictures how the patients included comprise a number of patients fulfilling the criteria for sit-stand test and a number of elderly, comorbid patients not fulfilling the criteria for sit-stand test that were tested outside the guideline

Data was collected on 160 patients with suspected COVID-19 triaged to ambulance response by general practioner between 6^th^ April 2020 and 6^th^ July 2020. Four patients were excluded (two due to missing identification and two were included twice). Thus, data on 156 unique patients were included in the study (Table 1). 86/156 patients were tested with the sit-stand test, but of these 30/86 (34.8%) were older than 70 years and/or had comorbidities for serious disease and were thus off-guideline tested. Figure 2 displays an overall overview of patients included in the study.

**Table 1:** Baseline characteristics of patients

	**Test performed *N* = 86**	**Test not performed *N* = 70**
**Age, median (IQR)**	**52** (35–64)	**70** (53–82)
**Male, n (%)**	**42** (48.8)	**42** (60.0)
**Fulfilled criteria for sit-stand test**^**a**^	56 (65.1)	
**Comorbidities, n (%)**		
Immunodeficiency	**1** (1.2)	**1** (1.4)
Severely increased blood pressure	**0** (0)	**0** (0)
Coronary artery disease	**8** (9.3)	**12** (17.1)
Cancer	**2** (2.3)	**11** (15.7)
Diabetes mellites with complications	**1** (1.2)	**5** (7.1)
Liver disease	**1** (1.2)	**1** (1.4)
Severe obesity (BMI 35–40)	**3 (**3.5)	**4** (5.7)
Chronic pulmonary disease	**11 (**12.8)	**21** (30.0)
Chronic kidney disease	**0** (0)	**4** (5.7)
Impaired coughing conditions	**0** (0)	**2** (2.8)
Pregnancy	**1** (1.2)	**0** (0)
**Covid-19 symptoms, n (%)**		
Coughing	**48** (48.8)	**31** (44.2)
Fever	**20** (23.3)	**30** (42.9)
Myalgia	**9** (10.5)	**6** (8.6)
Fatigue	**6** (7.0)	**9** (12.9)
Dyspnea	**37** (43.0)	**38** (54.3)
**Positive covid-19 test**^**b**^**, n (%)**	**6** (7.0)	**4** (5.7)

^a^ < 70 years of age with no risk factors for serious disease

^b^Positive COVID-19 test within 14 days prior to and 30 days after inclusion

### Test results and patient outcome

In total, 17/86 (19.7%) of patients tested with the sit-stand test decompensated during the test. Of patients decompensating during the sit-stand test, 9/17 (52.9%) were treated with oxygen within 7 days vs 2/69 (2.9%) of patients not decompensating during the test (*p* < 0.001) (Table 2).

**Table 2:** Outcome measurements for patients with sit-stand test performed (*n* = 86)

	**Decomp + ** **N = 17**	**Decomp –** **N = 69**
**Triage**		
Hospital admission, n (%)	**13** (76.5)	**12** (17.4)
ED but not admitted, n (%)^a^	**4** (23.5)	**7** (10.1)
Treated and released on-scene, n (%)	**0** (0.0)	**50** (72.5)
**Length of stay, index admission (days), mean (min–max)**	**3.5** (1–16)	**1.8** (1–7)
**Later admission within 7 days, n (%)**^**b**^	**0**(0.0)	**7** (10.1)
**Oxygen treatment**		
During index admission, n (%)	**9** (52.9)	**0** (0.0)
Later, within 7 days, n (%)**	**0** (0.0)	**2** (2.9)
**Intensive care unit admission, n (%)**		
During index admission, n (%)	**0** (0.0)	**0** (0.0)
Later, within 7 days, n (%)**	**0** (0.0)	**1** (16.7)
**Death within 30 days, n (%)**	**1** (5.9)	**0** (0.0)
**Positive covid-19 test**^**c**^**, n (%)**	**1** (5.9)	**5** (7.2)

Decomp + : patients who decompensated during the one-minute sit-stand test

Decomp -: patients who did not decompensate during the one-minute sit-stand test

^a^Seen in the emergency department, but not admitted to hospital

^b^Hospital admissions later than an index admission

^c^Positive COVID-19 test within 14 days prior to and 30 days after inclusion

In the group of patients who were off-guideline tested (i.e. > 70 years of age and/or with comorbidities), 11/30 (36.7%) decompensated during the test.Of these, 8/11 (72.7%) were treated with oxygen within 7 days compared to 0/19 (0%) in the group of patients not decompensating (*p* < 0.001). In the group of patients originally targeted in the guideline (i.e. < 70 years of age with no comorbidities) 6/56 (10.7%) decompensated during the test- Of these patients, 1/6 (16.7%) were treated with oxygen within 7 days compared to 2/50 (4%) among those not decompensating (*p* < 0.193).

## Discussion

This prospective cohort study investigated results of a prehospital one-minute sit-stand test for identifying patients in risk of deterioration among seemingly stable patients with suspected COVID-19 infection triaged by telephone consult by a GP to an ambulance response. The study demonstrated that twenty percent of patients decompensated during the test and of these patients, 53% were treated with supplementary oxygen within 7 days compared to 3% patients in the group of patients, who did not decompensate during the test. However, a large percentage of elderly patients with risk factors for serious disease were off-guideline tested with the sit-stand test and interestingly, the difference was only significant in this group of patients.

To our knowledge and findings, no other studies have investigated exercise testing in an emergency setting. Functional capacity tests such as cardiopulmonary exercise testing (CPET) have been widely used in patients suffering cardiopulmonary diseases to reveal oxygen desaturation and monitor disease progression and is considered gold standard in measure of exercise capacity [20]. Pulmonary diseases can be evaluated with the six minutes walk test (6MWT) which is well etasblished, easily performed and used in patients suffering chronic obstructive pulmonary disease. However both of these exercise test require either equipment or walking distance to be performed, which often limits their use in private homes or out-of-hospital use. The sit-to-stand test has therefore been evaluated by Ozalevli et. al and Crook et. al. as a less extensive alternative. They the found the sit-to-stand test to be well correlated with the 6MWT [9, 21].

Other studies have addressed the challenge of predicting outcome in COVID-19, and found that no clinical characteristics can isolated predict outcome [22]. Guo et al. retrospectively examined the ability of an early warning score including age, chronic diseases, neutrophil/leucocyte ratio, C-reactive protein and D-dimer to predict the clinical course of patients with COVID-19. By stratifying patients into low-, medium- and high risk groups they found significant difference in incidence of deterioration [23]. Prehospital use of this score would require extensive use of point-of-care diagnostics not presently available in most settings.

### Strength and limitations

The Danish Civil Registration System and PPJ ensure comprehensive data collection and allows the investigator to collect high-quality both pre- and in-hospital information regarding patient treatment and outcome, which is an important strength. The pragmatic implementational approach that highly reflects daily clinical practice during the COVID-19 pandemic is also a strength. However, 35% of patients with risk factors for serious disease were off-guideline tested with the sit-stand test. Inspired by Marino et al. we implemented the sit-stand test as a standard procedure with video-instructions and decision tools through usual communication platforms to all ambulance personell [24], but the rapid development of the ongoing pandemic limited the possibility for ongoing education and follow up. As a consequence, off-guideline use of the sit-stand test was frequent in a group of elderly comorbid patients. In addition, the low prevalence of COVID-19 makes the studied group of patients very heterogenous and the findings of the study are not generalizable to "suspected COVID-19" patients in high-incidence parts of the world. This affects the external validity of the results in relation to COVID_19. However, the results warrants future studies on the value of the sit-stand test as a more general predictive tool for decision-making.

The primary end-point, supplemental oxygen treatment within 7 days, may have been underestimated in the group of patients not decompensating during the sit-stand test, as they were less likely to be admitted to hospital. This may have falsely increased the predictive potential of the sit-stand test.

## Conclusion

In a population suspected of COVID-19, but with low COVID-19 prevalence, decompensation with the one-minute sit-stand test was observed in 20% of patients and was associated with oxygen treatment within 7 days. These findings generate the hypothesis that physical exercise testing may be usefull for decision making in emergency settings.

## Supplementary Information

**Additional file 1.**

## Data Availability

The dataset generated and analyzed during the current study is not public available due to sensitive personal data (civil registration number). Data is available from corresponding author upon reasonable request in anonymized form.
