# Supplementary material for: Implementation and findings on a one-minute sit-stand test for prehospital triage in patients with suspected COVID-19—a pilot project
Source: BMC Emerg Med. 2022 Mar 31;22:54. doi: 10.1186/s12873-022-00605-9 (PMC8968777; doi:10.1186/s12873-022-00605-9)
Supplement: Supplementary file 1 — Additional file 1. [file 12873_2022_605_MOESM1_ESM.docx]

| Appendix 1: Admission overview in patients who did not decompensate and were treated and released on-scene | | | | | | | | |
| --- | --- | --- | --- | --- | --- | --- | --- | --- |
| Record id | **Age (years)** | **Gender** | **Cause of readmission** | **Discharge diagnosis** | **Days after treated and left** | **Length of stay** | **Treatment** | **ICU** |
| Patients without risk factors | | | | | | | | |
| 6 | 52 | Female | Dyspnea | No clear diagnosis, no infection | 2 | 1 | Oxygen | No |
| 70 | 53 | Male | Acute abdominal pain | Appendicitis and peri-appendicular abscess | 1 | 4 | Antibiotics  Drainage tube | No |
| 139 | 49 | Male | Chest pain, myalgia | Perimyocarditis | 0 | 3 | KAG | No |
| 141 | 51 | Male | Diarrhea and vomiting | Debut of ulcerative  colitis | 1 | 7 | Antibiotics | No |
| 146 | 53 | Male | Obstipation and nausea | Covid-19 | 7 | 24 | Oxygen  Antibiotics  Intubation | Yes |
| 150 | 58 | Female | Myalgia, fever | Bacterial throat infection | 0 | 2 | Antibiotics | No |
| Patients with risk factors | | | | | | | | |
| 88 | 23 | Female | Pregnancy, dyspnea | Covid-19 | 2 | < 24 hours | Anti-coagulation | No |
